# A rare case of pulmonary nocardiosis comorbid with Sjogren’s syndrome

**DOI:** 10.1002/jcla.23902

**Published:** 2021-08-21

**Authors:** Yumeng Peng, Xiaoyan Dong, Yongze Zhu, Huoyang Lv, Yumei Ge

**Affiliations:** ^1^ Center of Clinical Laboratory Medicine the Zhejiang Provincial People’s Hospital People’s Hospital of Hangzhou Medical College Zhejiang China; ^2^ Bengbu Medical College Anhui China; ^3^ The Second Medical College Zhejiang Chinese Medical University Zhejiang China

**Keywords:** etiological examination, next‐generation sequencing, *Nocardia terpenica*, pneumonia, Sjogren's syndrome

## Abstract

**Background:**

Nocardia is an opportunistic pathogen, which occurs in patients with autoimmune diseases and immune dysfunction, and can cause bacteremia and other life‐threatening complications. The clinical manifestations of Nocardia pneumonia are similar to tuberculous and other clinical common bacterial pneumonia, but its antibacterial treatments are different and detection methods are unique, which may lead patients to suffer for many years due to clinical misdiagnosis and missed diagnosis.

**Methods:**

Imaging and laboratory examinations were performed for preliminary diagnosis, and next‐generation sequencing was used to identify the exact species type of *Nocardia* in the bronchoalveolar lavage fluid (BALF) of the patient.

**Results:**

Imaging and laboratory parameters preliminarily implied that the patient was infected with Nocardia with Sjogren's syndrome (SS), and NGS showed that the strain was *N*. *terpenica*.

**Conclusions:**

Accurate etiological diagnosis and corresponding antibiotics are key to improve the prognosis of pulmonary nocardiosis in this case. Nocardia pneumonia is rare in clinical practice; it is of great medical significance to improve the understanding of pulmonary nocardiosis.

## 1 INTRODUCTION

Nocardia is Gram‐positive, weakly acid‐fast, catalase‐positive, nonmotile, branching rod‐shaped aerobic bacteria, belonging to actinomycetales.^1^, ^2^ Nocardia widely exists in soil, water, air, and rotten plants. It is common in patients with T‐cell deficiency (leukemia or AIDS), long‐term usage of immunosuppressants (such as organ transplantation and malignant tumor), or patients with underlying diseases (such as diabetes and chronic kidney disease).^3^, ^4^, ^5^, ^6^ Pulmonary nocardiosis is a purulent and granulomatous disease caused by *Nocardia* invading the lung through respiratory tract inhalation or skin lesions. It can spread into the brain, kidney, and other organs by blood dissemination and even form life‐threatening sepsis.

The clinical manifestations of pulmonary nocardiosis are fever, cough, expectoration, dyspnea, chest pain, hemoptysis, weight loss, fatigue, and other symptoms. Pulmonary nocardiosis is commonly characterized by infiltrative lesions, cavities, nodules, or masses in lung segments or lobes in computed tomography. Because its clinical manifestations and imaging are not specific to common bacterial infection, pulmonary aspergillosis, lung cancer, pulmonary abscess, and tuberculosis, it often leads to misdiagnosis and missed diagnosis.^7^ What is worse, than other bacteria, *Nocardia* grows slowly.^8^ After 7 days of aerobic culture at 37 ℃, the wrinkled milky white colonies embedded in the culture medium can be observed. Therefore, it is easy to be missed in the laboratory diagnosis process. Diagnosis and treatment may be delayed due to the late results of pathogen identification. We describe a patient with pulmonary nocardiosis comorbid with SS with hemoptysis for more than 20 years.

## 2 CASE PRESENTATION

### 2.1 Clinical features

A 70‐year‐old male patient was admitted because of hemoptysis for more than 20 years without obvious inducement. The patient had a history of drinking and smoking for 50 years, about 500 ml of wine, and 20 cigarettes a day. He presented with bright red blood, cough and expectoration, no chest tightness, chest pain, shortness of breath, chills, or fever. Chest CT from the local hospital showed multiple nodules and bronchiectasis in both lungs. He did not have any treatment in the local hospital and came to Zhejiang Provincial People's Hospital (Zhejiang province, China) for further diagnosis. Later, he was treated with pituitrin and tranexamic acid injection for hemostasis, ceftriaxone sodium for anti‐infection, and ambroxol for resolving phlegm. His condition improved while he still had repeated intermittent hemoptysis for last 2 years, with small amount, bright red blood, cough and expectoration, dark brown sputum, and occasional active chest tightness.

### 2.2 Imaging examination

**FIGURE 1:**
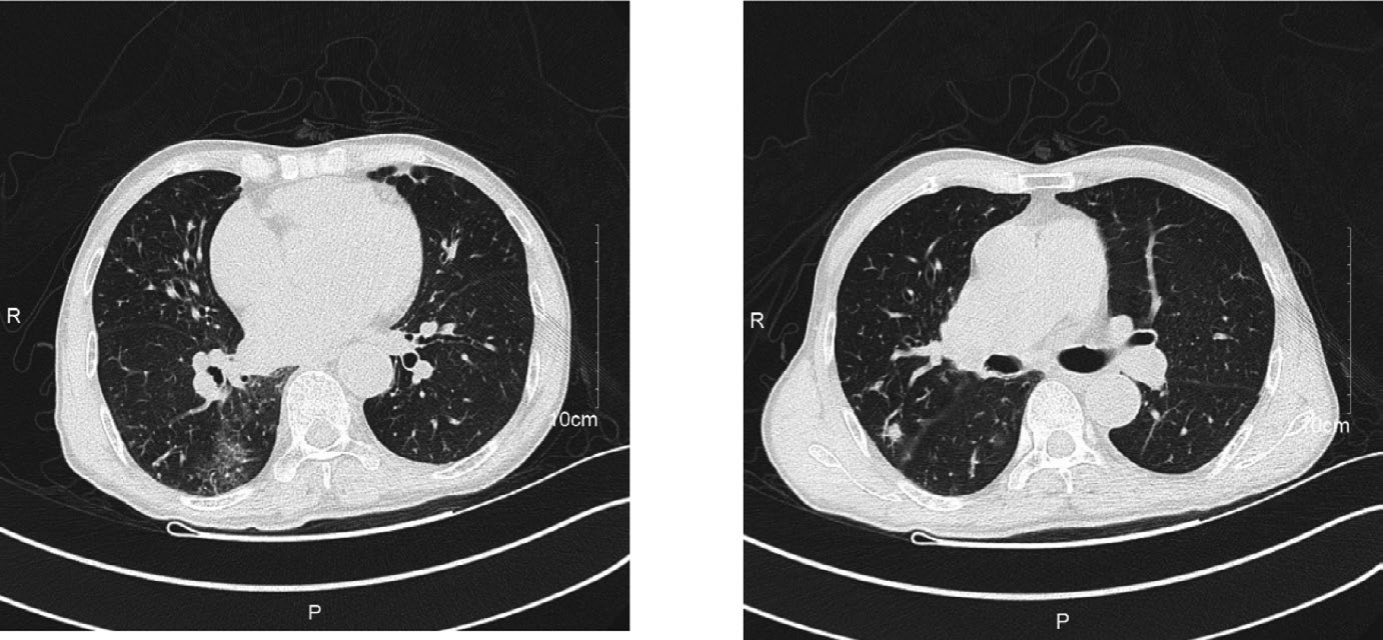
High‐resolution computed tomography (HRCT) of the thorax showed bronchiectasis and inflammation involving in both lungs. Patchy and nodular high‐density shadows were scattered in both lungs, with unclear margin. Bilateral bronchiectasis and wall thickening were observed. Nodular high‐density shadows were found in local lumen, especially in right lung. The right upper lobe (Se4, im69) showed nodular high‐density shadow with unclear edge, about 9 mm in diameter. Small nodules and dense foci were seen in the right lung. In addition, small cystic areas without lung markings were found in both lungs, with clear edges

High‐resolution computed tomography (HRCT) showed that the nodules in the upper lobe of the left lung were newly found and could not currently completely exclude tumor lesions. There were multiple bronchiectasis and some bronchial mucus embolism in both lungs. Inflammatory foci were scattered in both lungs (Figure 1). Therefore, doctors suggested improving laboratory examination to differentially diagnose between pulmonary tuberculosis and lung cancer. The patient was recommended to review and further examination such as acid‐fast staining of sputum and tuberculin purified protein derivative (PPD) test after anti‐inflammatory treatment.

### 2.3 Immunologic examination

The main serological abnormalities were antinuclear antibody (ANA)‐positive (++, 1:320); the immunofluorescent pattern of ANA was granule type and cytoplasmic granule type; antinuclear extract antibody (ENA)‐positive (+); anti‐SSA (60 KD) antibody‐positive (+++); anti‐Ro antibody (52 KD)‐positive (+++); anti‐histone antibody weakly positive (±), Ig G (20.30 g/L, the normal reference interval was 7.51–15.6 g/L) and Ig E (123.0 IU/ml, the normal reference interval was 0−87 IU/ml) increased significantly, ANCA was negative. The abnormal results were consulted by the rheumatology department and ophthalmology department, combined with tear secretion test. The tear film rupture time was 5S/7S. The final diagnosis was SS, involving both eyes.

### 2.4 Hematologic examination

The increase in inflammatory indexes in peripheral blood suggested that there might be infection in the patient. The main abnormal results were increases in white blood cell count (13.01 × 10^9^/L), neutrophil proportion (86.6%), high sensitivity C‐reactive protein (87.2 mg/L), and erythrocyte sedimentation rate (62 mm/h) and decreases in albumin (36.2 g/L). Therefore, the doctor gave the patient 100 ml levofloxacin injection containing 0.5 g levofloxacin and 0.9 g sodium chloride every day for anti‐infection treatment and suggested etiological examination for further diagnosis.

### 2.5 Histopathology and cytology examination

**FIGURE 2:**
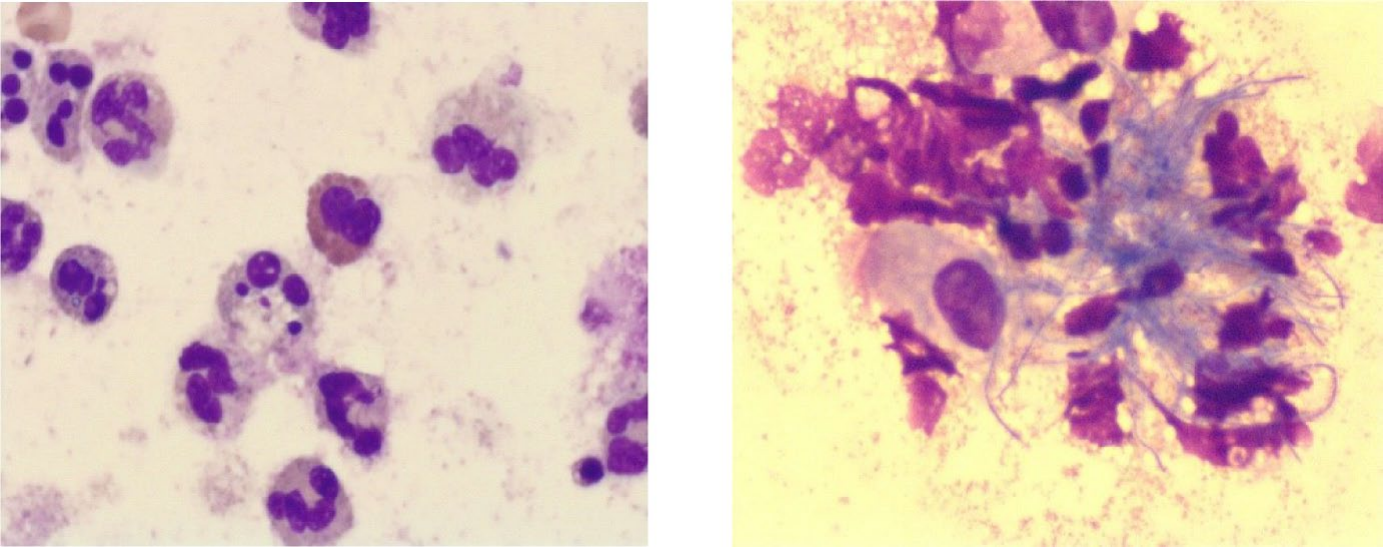
The results of cellular report of alveolar lavage fluid. There were many nucleated cells, mainly neutrophils, macrophages, lymphocytes, eosinophils, hemosiderin granules, radial rod‐shaped bacteria, and no fungi or other special abnormal cells were found in alveolar lavage

Cytopathological examination showed that no cancer cells were found in brush biopsy of the middle lobe of the right lung. But the high proportion of neutrophils in BALF indicated that there might be acute inflammatory infection, and it was necessary to pay attention to the pile of radial rod‐shaped bacteria (Figure 2).

### 2.6 Etiological examination

**FIGURE 3:**
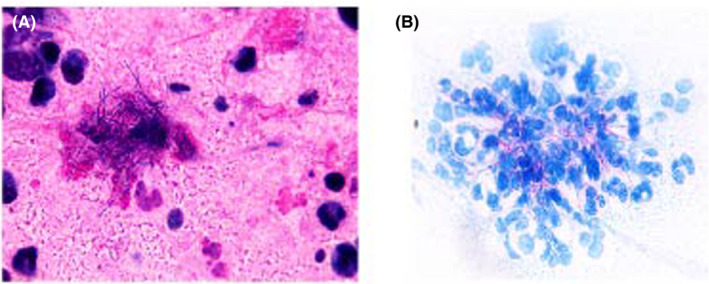
(A) Gram‐positive and slender branching rods initially considered as Nocardia. Sputum specimen was Gram‐stained and examined under an oil immersion microscope at ×1000 magnification. (B) Acid‐fast branching rods on Ziehl‐Neelsen stain of sputum smear examined under an oil immersion microscope at ×1000 magnification. Weakly acid‐fast, filamentous bacteria with branched vegetative hyphae could be clearly observed in the blue background

No acid‐fast bacilli were found in the smear of acid‐fast staining of the smears of transcatheter bronchial brushing. No fungi were observed under an ultraviolet microscope after the fungal fluorescence staining of BALF. The tuberculosis DNA of sputum detected by fluorescence PCR was negative (−). In the process of smear for acid‐fast bacilli in clinical laboratory, Gram‐positive, weakly acid‐fast, and filamentous bacilli were found and were highly suspected to be *Nocardia* (Figure 3A,B).

### 2.7 NGS analysis

Sample processing and nucleic acid extraction were according to the steps of the TIANamp Micro DNA Kit (DP316, TIANGEN BIOTECH). 600 μL sputum or BALF of the patient was mixed and shaken with glass beads, and the extracted DNA was sonicated to fragments with a size of 100–150 bp. NGSmaster fully automated library construction and MGIseq‐2000 were used for sequencing and analyzing. DNA fragments were amplified by terminal repair, ligation, and nonbiased PCR. The size of inserted fragments (200–300 bp) was determined by Agilent 2100 Bioanalyzer quality control library, and the concentration of DNA library (>2 ng/µl) was determined by using a qubit dsDNA HS assay kit (Thermo Fisher Scientific Inc.). The qualified library sequences were cyclized to form a chain ring structure. After cyclization, DNB nanospheres were generated by rolling ring replication (RCA). The prepared DNB nanospheres were loaded into the sequencing chip and sequenced by MGIseq‐2000. Sample results were compared with a microbiological database including pathogenic bacteria (6350 species), fungi (1064 species), viruses (4945 species), and parasites (234 species) to obtain the number of sequences that could be matched to a certain pathogen. The results showed that it was *N*. *terpenica* with a sequence number of 306 and a 90.53% relative abundance. The patient's laboratory test results are shown in Tables 1 and 2.

**TABLE 1:** Clinical parameter detection of peripheral blood of the patient

Clinical laboratory test	Reference value of normal subjects	Unit	Patient
Perinuclear‐ANCA	Negative (<1:3.2)		Negative (−)
PR3‐ANCA	Negative (<1:3.2)		Negative (−)
Untypical ANCA	Negative (<1:3.2)		Negative (−)
Anti‐endothelial cell antibodies	Negative (<1:32)		Negative (−)
Anti‐proteinase 3 antibody	0.0–20.0	RU/ml	2.5
Anti‐MPO antibody	0.0–20.0	RU/ml	1.8
Anti‐glomerular basement membrane Antibody (anti‐GBM)	0.0–20.0	RU/ml	0
SCC	0–3	ng/ml	1.6
BNP	0–160	pg/ml	10.1
cTnI	0–0.05	µg/L	0.045
Total triiodothyronine (TT3)	0.66–1.61	µg/L	1.15
Total thyroxine (TT4)	54.4–118.5	µg/L	91.43
Thyroid stimulating hormone (TSH)	0.34–5.6	mIU/L	5.51
Free triiodothyronine (FT3)	2.14–4.21	ng/L	3.28
Free thyroxin (FT4)	5.9–12.5	ng/L	7.43
Thyroglobulin antibody (TGA)	<4.00	IU/ml	<0.9
Thyroid peroxidase antibody (TPO)	<9.00	IU/ml	2.5
Neuron special endolase (NSE)	0.0–16.3	ng/ml	11.1
Procalcitonin PCT	0.00–0.25	ng/ml	0.02
AFP	0.0–20.0	µg/L	4
Carcinoembryonic antigen (CEA)	0.0–5.0	µg/L	1.9
Carbohydrate antigen 12‐5 (CA12‐5)	0.0–35.0	U/ml	34.1
Carbohydrate antigen 19‐9 (CA19‐9)	0.0–37.0	U/ml	28.4
Carbohydrate antigen 15‐3 (CA15‐3)	0.0–28.5	U/ml	16.2
Total PSA	0.00–4.00	µg/L	0.72
Cytokeratin19	0.0–3.8	ng/ml	2.5
Carbohydrate antigen 72‐4 (CA72‐4)	0.0–19.3	U/ml	3.3
Progastrin releasing peptide, ProGRP	25.0–78.0	pg/ml	39.7
Immunoglobulin G (IgG)	7.51–15.60	g/L	20.3
Immunoglobulin E (lgE)	0–87	IU/ml	123
Immunoglobulin A (IgA)	0.82–4.53	g/L	2.01
Immunoglobulin M (IgM)	0.46–3.04	g/L	0.69
Complement C3	0.79–1.52	g/L	1.01
Complement C4	0.16–0.38	g/L	0.24
Rheumatoid factor	0.0–20.0	IU/ml	<20
Antinuclear antibody	Negative (<1:32)		Positive (++ 1:320)
Types of antinuclear antibody			颗粒型+胞浆颗粒
Anti‐ds‐DNA qualitative	Negative (<1:3.2)		Negative (−)
Anti‐ds‐DNA quantitative	0.0–75.0	IU/ml	8.2
Antinuclear extract antibody	Negative (−)		Positive (+)
Anti‐nucleosome antibody (AnuA)	Negative (−), 0–15		Negative (−) 0
Anti‐Sm antibody	Negative (−), 0–15		Negative (−) 0
Anti‐U1RNP antibody	Negative (−), 0–15		Negative (−) 8
Anti‐SSA antibody	Negative (−), 0–15		Positive (++) 31
Anti‐R0 antibody	Negative (−), 0–15		Positive (+++) 52
Anti‐SSB antibody	Negative (−), 0–15		Negative (−) 2
Anti‐scl‐70 antibody	Negative (−), 0–15		Negative (−) 2
Anti‐PM‐Scl antibody	Negative (−), 0–15		Negative (−) 1
Anti‐Jo‐1 antibody	Negative (−), 0–15		Negative (−) 1
Anti‐histone antibody	Negative (−), 0–15		Positive (+−) 20
Antiproliferative extractable nuclear antigens	Negative (−), 0–15		Negative (−) 10
ARPA/Rib‐P	Negative (−), 0–15		Negative (−) 1
Anticentromere antibody	Negative (<1:32)		Negative (−)
Centromere antibody (Cenp B)	Negative (−), 0–15		Negative (−) 1
Anti‐mitochondrial antibody	Negative (<1:32)		Negative (−)
Mitochondria antibodyM2	Negative (−), 0–15		Negative (−) 2
Antinuclear membrane antibody	Negative (<1:32)		Negative (−)
Anti‐NOR antibody	Negative (<1:32)		Negative (−)
Anti‐actin antibody	Negative (<1:32)		Negative (−)
Microtube spindle antibodies	Negative (<1:32)		Negative (−)
Nucleolinus antibody	Negative (<1:32)		Negative (−)
Anticentriole antibody	Negative (<1:32)		Negative (−)
Anti‐chromosome antibody	Negative (<1:32)		Negative (−)
Antinuclear skeleton protein antibody	Negative (<1:32)		Negative (−)
Normal control value PT		Seconds	11
Prothrombin time	9.3–12.6	Seconds	11.8
International normalized ratio	0.85–1.20		1.08
Normal control value appt	22.7–34.1	Seconds	26.2
Partial thromboplastin time	21.0–32.8	Seconds	25.4
Partial thromboplastin time Ratio	0.80–1.20		0.92
Normal control value TT		Seconds	17.1
Thrombin time	13.7–20.5	Seconds	18
Thrombin time ratio	0.80–1.20		0.92
Fibrinogen	2.00–4.00	g/L	4.62
D‐dimer	0.0–550.0	µg/L	430
Total protein	65–85	g/L	74.3
Albumin	40–55	g/L	38.3
Globulin	20–40	g/L	36
Albumin globulin ratio	1.2–2.4		1.06
ALT	9–50	U/L	8
Aspartate aminotransferase (AST)	15–40	U/L	24
AST:ALT	0.45–1.80		3
γ glutamyltranspeptidase (γ GT)	10–60	U/L	30
Alkaline phosphatase	45–125	U/L	103
Total bilirubin	3.4–24	µmol/L	7.3
Direct bilirubin	0.0–6.8	µmol/L	1.4
Indirect bilirubin	1.7–17.2	µmol/L	5.9
Total bile acid	0.0–15.0	µmol/L	2
Glucose	3.92–6.16	mmol/L	3.99
Urea	3.10–8.00	mmol/L	8.5
Creatinine	44.0–133.0	µmol/L	69.2
Urea nitrogen:creatinine	7.5–27.5		30.71
Uric acid	208–428	µmol/L	375
Potassium	3.5–5.3	mmol/L	3.65
Sodium	137–147	mmol/L	141.9
Chlorine	99–110	mmol/L	107.9
Calcium	2.11–2.52	mmol/L	2.19
Magnesium	0.75–1.02	mmol/L	0.91
Phosphorus	0.85–1.51	mmol/L	1.25
Osmotic pressure	275–300	mosm/L	285
Total cholesterol	3.11–5.96	mmol/L	5.04
Triglyceride	0.34–1.70	mmol/L	0.97
High‐density lipoprotein cholesterol	1.04–2.05	mmol/L	1.24
Low‐density lipoprotein cholesterol	2.10–3.10	mmol/L	3.15
Non‐high‐density lipoprotein cholesterol	0.86–4.1	mmol/L	3.8
Apolipoprotein A	1.2–1.8	g/L	1.38
Apolipoprotein b	0.6–1.14	g/L	1.01
APOB/APOA	0.4–1.1		0.73
Serum‐free fatty acid	129–769	µmol/L	526
Lactate dehydrogenase	120–250	U/L	167
Lipoprotein A	0–300	mg/L	76
Creatine kinase	50–310	U/L	66
Cholinesterase	4500–13,000	U/L	8396
Dipeptidyl peptidase Ⅳ	44–116	U/L	53
α‐l‐fucosidase	0–40	U/L	29
Homocysteine	0.0–20	µmol/L	17.1
WBC count	3.5–9.5	×10^9^/L	13.01
Lymphocyte classification	20–50	%	7.8
Monocyte classification	3–10	%	4.3
Neutrophil classification	40–75	%	86.6
Eosinophil classification	0.4–8.0	%	1.1
Basophil classification	0–1	%	0.2
Lymphocyte count	1.1–3.2	×10^9^/L	1
Monocyte count	0.1–0.6	×10^9^/L	0.6
Neutrophil count	1.8–6.3	×10^9^/L	11.3
Basophil count	0–0.06	×10^9^/L	0.03
Eosinophil count	0.02–0.52	×10^9^/L	0.14
Red blood cell count	4.3–5.8	×10^12^/L	4.4
Hemoglobin	130–175	g/L	135
Hematocrit	0.40–0.50		0.411
Mean corpuscular hemoglobin	27–34	pg	30.7
Mean corpuscular hemoglobin concentration	316–354	g/L	328
Red blood cell distribution width	11.6–14.8	%	13.6
Platelet count	125–350	×10^9^/L	197
Platelet hematocrit	0.1–0.25	%	0.2
Platelet large cell ratio	13.0–43.0	%	26.3
Erythrocyte sedimentation rate	0–43	mm/h	62

**TABLE 2:** Serum‐ infected markers of the patient

Clinical laboratory test	Reference value of normal subjects	Patient	Unit
HBsAg	Negative (−)	Negative (−)	
Hepatitis B surface antibody	Negative (−)	Negative (−)	
HBeAg	Negative (−)	Negative (−)	
HBeAb	Negative (−)	Negative (−)	
Anti‐HBc	Negative (−)	Negative (−)	
HCV antibody	Negative (−)	Negative (−)	
HIV‐1+2 antibody+P antigen	Negative (−)	Negative (−)	
TRUST	Negative (−)	Negative (−)	
TP‐Ab	Negative (−)	Negative (−)	
MP‐IgM	Negative (−)	Negative (−)	
DNA of *Mycobacterium tuberculosis*	Negative (−)	Negative (−)	
Galactomannan (GM)	Negative (−)	Negative (−)	
1‐3 ‐ β ‐ D glucan	0–60	58.13	pg/ml
Antistreptolysin‐O (ASO)	0.0–116.0	74.5	IU/ml
High‐sensitivity C‐reactive protein	0.0–8.0	87.2	mg/L

### 2.8 Treatment and prognosis

The doctor decided to stop levofloxacin intravenous drip QD (once a day), instead of isepamicin 0.4 g with sodium chloride 250 ml intravenous drip QD, continue to give cefmetazole sodium 2g intravenous drip BID (twice a day) for anti‐infection, plus sulfamethoxazole tablets (SMZ) for treatment of *Nocardia* infection. Vitamin B6 10 mg oral QD, vitamin‐B1 10 mg oral TID (three times a day), sodium bicarbonate 1 g oral TID for alkalization of urine, and compound sulfamethoxazole 1.44 g oral TID for anti‐inflammatory. After a week of medication, the patient's cough and expectoration improved, no chest tightness, shortness of breath, no fever and chills, the breath sounds of both lungs were thick, no obvious rhonchus and moist rales were heard, the rhythm was regular, and no obvious pathological murmur was heard in the auscultation area of each valve. Experts agreed that the diagnosis of *Nocardia* infection was clear, and the treatment of the patient was effective.

## 3 DISCUSSION

The clinical manifestations of nocardiosis are similar to fungal, mycobacterium and bacterial infection, and lung adenocarcinoma. Nocardia can cause granuloma, abscess, and pulmonary nodules. The main respiratory symptoms are cough, expectoration, hemoptysis, chest pain, fever, dyspnea, fatigue, and empyema. Chest CT and other imaging findings are more than medium density of patchy infiltration, nodules, lung abscess, cavity, and hilar lymph node enlargement. Therefore, pulmonary nocardiosis is often misdiagnosed before definite etiological examination results have been obtained.^9^, ^10^

For infected patients, timely ascertaining of pathogenic bacteria is important for effective treatment. It can reduce the rate of mortality and readmission and shorten the length of hospitalization. In this case, the patient was elderly and immunocompromised with SS and chronic diseases of bronchiectasis and hemoptysis that might be often attacked by more than one pathogen. NGS helped clinicians quickly and accurately find the specific pathogens of infection and exclude other bacterial co infection, which played a decisive role in the selection of appropriate antimicrobial therapy. We used NGS to determine the exact species type of *Nocardia* within one day, and he was diagnosed with infection of *N*. *terpenica*, which was rarely documented elsewhere. Thus, SMZ was used for anti‐infection treatment according to the NGS result of Nocardia, which showed notable curative effectiveness.

NGS has been applied in the pathogenic diagnosis of diseases because it is more sensitive than 16S rDNA sequencing analysis in the diagnosis of unspecified pathogens, especially rare pathogens. It can detect almost all kinds of pathogens in the samples, while 16S rDNA sequencing technique can only detect a specific target pathogen that matches the corresponding primers and rare pathogens may be missed, or primers containing mismatches with the tested pathogens may be used, resulting in detection failure and reduced sensitivity.^11^

Cases of *N*. *terpenica* infection are rare in clinic. *Nocardia* is an opportunistic pathogen and usually infected in immunocompromised patients.^12^, ^13^, ^14^, ^15^ Patients with a history of solid organ transplantation, hematopoietic stem cell transplantation, malignant tumor, HIV infection, and chronic glucocorticoid therapy are often at higher risk.^16^, ^17^, ^18^, ^19^ This article reported a case of pulmonary nocardiosis comorbid with SS that has been rarely reported before. SS is a chronic inflammatory disease mediated by autoimmunity, which is characterized by infiltration of T cells, B cells, macrophages, and other immune cells in exocrine glands such as salivary gland and lacrimal gland, accompanied by clinical manifestations such as keratoconjunctivitis sicca and xerostomia.^20^ There are antinuclear antibody, anti‐SSA antibody, anti‐SSB antibody, antiplatelet antibody, antiglobulin antibody, and other autoantibodies in the serum and usually accompanied by hyperimmunoglobulinemia.^21^, ^22^ In our case, the patient with SS was confirmed to have abnormal immune function according to immunological test results and clinical manifestations, which might be one of the inducing factors of *Nocardia* infection.

The course and prognosis of infectious diseases are closely related to the pathogenicity of pathogens and are more important to the body's own immune function against infection. Patients with autoimmune diseases such as SS and abnormal immune function usually cannot induce and produce effective protective antibodies after *Nocardia* infection. Those patients often have a higher risk of reinfection and preventive antibacterial treatment may be necessary. What is worse, the use of immunosuppressive drugs for autoimmune diseases or tumors will often aggravate *Nocardia* infection. Therefore, we should pay more attention to *Nocardia* infection in patients with immunodeficiency or chronic underlying diseases.

## 4 CONCLUSION

The clinical manifestations of Nocardia infection are not characteristic, and such cases are rarely reported. Thus, pathogenic detection has irreplaceable significance in the diagnosis of pulmonary nocardiosis. The possibility of *Nocardia* should be considered by the technical personnel of clinical laboratory when they observe weak acid‐fast, fibrinous bacteria with branched vascular hyphae in sputum samples. NGS has unique advantages in determining the types of pathogens in samples and identifying rare pathogens such as *Nocardia*, which is worthy of promotion and application in clinical diagnosis. In addition, *Nocardia* infection with autoimmune diseases in patients with clinical medication and anti‐infection therapy has particularity. We should eliminate *Nocardia* infection first and then use the treatment for their underlying diseases and prophylactic anti‐infection also should be considered later.

## 5 HIGHLIGHT

(1) Pulmonary nocardiosis comorbid with Sjogren's syndrome was rare in clinic, and its diagnosis is challenging. (2) NGS is more sensitive than 16S rDNA sequencing technique in the diagnosis of unspecified pathogens and can detect almost all kinds of pathogens in the samples. *Nocardia terpenica* was ascertained in BALF of this patient by NGS. (3) Patients with autoimmune diseases such as Sjogren's syndrome should be given more attention for anti‐*Nocardia* infection.

## CONFLICT OF INTERESTS

The authors declare that they have no competing interests.

## AUTHOR CONTRIBUTIONS

YMP collect the patient clinical information. YMP, XYD, YZZ, and HYL analyzed the data. YMP and YMG drawn the manuscript. All authors read and approved the final manuscript.

## CONSENT FOR PUBLICATION

Written and informed consent was obtained from the patient for publication of this case report and any accompanying images.

## Data Availability

The datasets used and/or analyzed during the current study are available from the corresponding author Yumei Ge on reasonable request.
